# Desminopathy: A Rare Cause of Cardiac Arrhythmia

**DOI:** 10.1016/j.jaccas.2026.109779

**Published:** 2026-08-10

**Authors:** Henry Ward, Hayley Nehoff, Ozayr Ameen, Ian G. Crozier

**Affiliations:** a Department of Cardiology, Christchurch Hospital, Christchurch, New Zealand; b Department of Neurology, Christchurch Hospital, Christchurch, New Zealand

**Keywords:** arrhythmia, cardiomyopathy, desmin, desminopathy, myopathy

## Abstract

**Background:**

Desminopathy is a disorder of the desmin intermediate filament network, caused by a defect in the desmin gene (*DES*), which can lead to cardiomyopathy and skeletal myopathy. However, cardiac arrhythmia is a rare presentation of desminopathy.

**Case Summary:**

A previously well 29-year-old woman presented with ventricular fibrillation and complete heart block, with subsequently slow decline in skeletal muscle function. Following extensive work-up, she was found to have a novel mutation of the desmin gene (*DES* c.640-1G>C).

**Discussion:**

We have identified a new *DES* variant possibly causing ventricular fibrillation, complete heart block, and skeletal myopathy. There is currently no cure, and management focusses on optimizing medical management and quality of life.

**Take-Home Messages:**

It is important to determine the cause for unusual arrhythmic presentations, and we hope to increase awareness of genetic causes for cardiac arrhythmias. Multidisciplinary teamwork is important in managing these cases and optimizing patient care.

## Visual Summary

**Figure:**
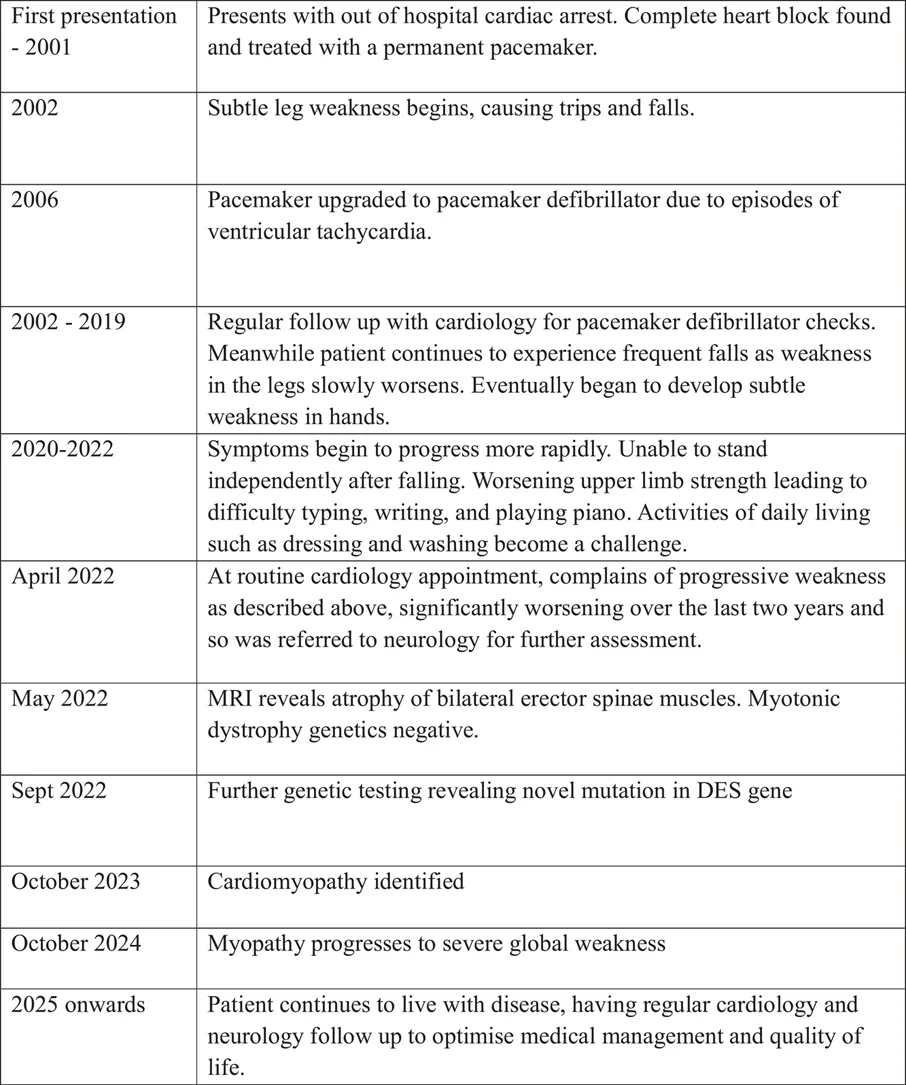

Desminopathy is a rare cause of cardiac arrhythmia with various manifestations, including myopathy, cardiomyopathy, arrhythmias, or a mixture of these, and it is caused by defects in the desmin gene (*DES*). This is located at chromosome 2q35 and contains 470 amino acids in 9 exons and encodes a class III intermediate filament specific to muscle called desmin.^1^ Desmin filaments form a network between cardiac and skeletal myocytes which is important in transmitting mechanical forces and maintaining the structure of myofibrils.^2^ In the heart, desmin is also present in key structures of the cardiac conduction system.^2^

## Case Presentation

A previously well, 29-year-old woman presented to hospital in 2001 with an out-of-hospital cardiac arrest with documented ventricular fibrillation. Following resuscitation, she had persisting complete heart block. There were no other extracardiac features, such as motor weakness, noted on examination.

## Investigations and Management

Coronary angiogram showed normal coronary arteries, and echocardiogram demonstrated preserved left ventricular function with an ejection fraction of 55%, though with midseptal dyskinesis and apical hypokinesis. The initial diagnosis was ventricular fibrillation secondary to heart block of unknown cause, and she was managed with implantation of a permanent pacemaker.

The patient's pacemaker was upgraded to an implantable pacemaker-defibrillator in 2006 following multiple episodes of symptomatic nonsustained ventricular tachycardia recorded on her device, and perceived risk of further ventricular arrhythmias and sudden death.

Since her initial cardiac presentation, the patient has experienced gradual decline in skeletal muscle strength. This resulted in her being referred for neurological review during which weakness was noted, most pronounced in the distal muscles initially, but gradually progressing proximally over the years to severe global weakness. Her most recent neurological examination demonstrated bilateral foot drop and profound weakness and muscle wasting in the proximal and distal upper and lower limbs, Medical Research Council (MRC) grade 2/5, with the exception of the quadriceps muscles which remained relatively preserved at MRC grade 4/5. Reflexes were generally preserved, symmetrical, and graded 1+. Sensation remained normal.

Nerve conduction studies revealed no sign of demyelination, while needle electromyography suggested a chronic myopathy without inflammation as the cause of weakness. A myositis screen was negative, and protein electrophoresis, C-reactive protein, immunoglobulins, and thyroid function test were all normal. Magnetic resonance imaging of her brain and spine was performed to evaluate for a central cause of her symptoms. The spinal cord was normal but the study had to be terminated before imaging of the brain could be obtained given patient claustrophobia. The scan also noted marked erector spinae and gluteus maximus fatty atrophy (Figure 1). Further genetic testing was arranged following this initial neurological assessment. Genetic testing of the *DMPK* (dystrophia myotonica protein kinase) gene was normal, excluding myotonic dystrophy type 1.

**Figure 1:**
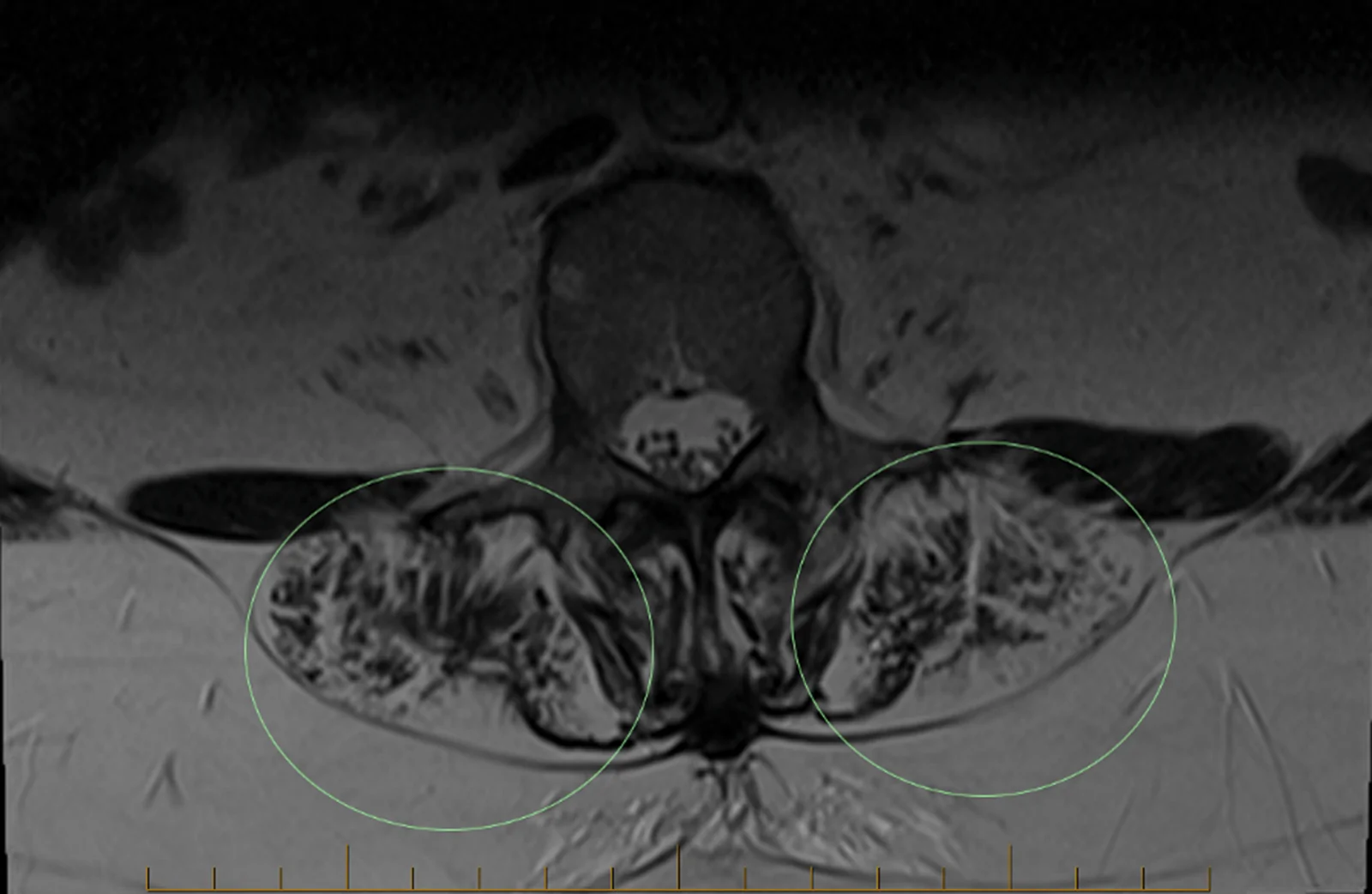
T2 Sequence MRI Image Depicting Axial Section of the Lumbar Spine at the Level of L4 The circled areas demonstrate extensive fatty infiltration and atrophy of bilateral erector spinae muscles. MRI = magnetic resonance imaging.

On further gene sequencing, however, they were found to be heterozygous for *DES* c.640-1G>C. This causes substitution of a nucleotide within a splice site and is predicted to cause in-frame skipping of exon 3. Many other genes were evaluated, excluding potential alternative diagnoses such as *LMNA* (lamin A/C) myopathy (Supplemental Appendix A).

Recently, the patient has also developed a dilated cardiomyopathy, left ventricular ejection fraction of 30%, and overt heart failure. Respiratory muscle function is also impaired, with diaphragmatic paralysis seen on imaging and a restrictive pattern on pulmonary function testing. She is currently asymptomatic of this.

## Outcome and Follow-Up

The patient continues to have regular follow-up with cardiology and neurology. There is no available cure for this condition, and the focus is on monitoring symptoms of cardiac dysfunction, progression of myopathy, and providing support for her to continue employment and maintain her quality of life.

## Differential Diagnosis

The differential diagnosis for her initial presentation of heart block included cardiac sarcoidosis, dystrophia myotonica, a *SCN5A* mutation, and later presentation of congenital complete heart block. Differentials for myopathies associated with arrhythmias include polymyositis, several muscular dystrophies, Andersen-Tawil syndrome, and Kearn-Sayre syndrome. *GNE* myopathy is another differential, and although cardiac muscle is not typically affected, cardiac involvement has been reported in some patients with *GNE* myopathy.^3^ It is a quadriceps-sparing myopathy, which is consistent with the neurological examination findings.

## Discussion

The desmin filament network is important in maintaining normal cell function and structural integrity.^1^^,^^2^ Mutations in *DES* can cause network disorganization or complete loss of function. These conditions are most commonly inherited in an autosomal-dominant pattern.^2^ Such cases are often slower in onset and progression, whereas the autosomal-recessive is typically more severe and manifests earlier. Sporadic cases such as ours are associated with de novo mutations and have a wide variability in severity.^4^

The mutation in this case resulted in nucleotide substitution within a splice site, likely causing abnormal splicing. This variant is predicted to cause in-frame skipping of exon 3, causing loss of 32 amino acids (residues 214-245). Functional analysis of desmin protein lacking exon 3 has shown disorganized distribution and aggregation of the protein.^5^^,^^6^ Mutations causing a splicing defect and exon 3 skipping has been shown to cause disease.^6^

Phenotypes associated with exon 3 mutations include arrhythmogenic right ventricular cardiomyopathy, atrioventricular block, and cardiomyopathy.^5^, ^6^, ^7^ Splice-site defects are relatively uncommon, with missense mutations being the most common cause of desminopathies.^2^ Clinical manifestations of missense, splice site, and other gene variants are varied and can present with isolated or combined skeletal and cardiac myopathy.^4^

In the present case, the primary manifestation of the variant was complete atrioventricular block, with the skeletal and cardiac myopathy developing gradually over time. In this case, diaphragmatic paralysis was only found because of an incidentally raised diaphragm on chest x-ray that prompted fluoroscopic investigation and pulmonary function testing. This suggests that clinicians could consider proactively monitoring for respiratory decline, as findings may manifest before symptoms develop.

The identified gene variant was initially classified as likely pathogenic by an independent laboratory (Supplemental Appendix B). However, subsequent analysis by another laboratory reclassified it as a variant of uncertain significance (VUS). Variants are evaluated based on evidence from population and gene-specific databases, and minority ethnicities may be under-represented. Our patient is Samoan, and it may be that a relative lack of available genetic data in this ethnic group has prevented the laboratory from confirming the variant as pathogenic or not. There is no available population frequency data for this variant in gnomAD, and there are no specific Pacific Islander controls. This is a potential challenge for investigators in the field working with minority groups such as Māori or Pacifica, as limited data contributes to genetic uncertainty, potentially leading to delays in diagnosis and treatment.

Given the genetic nuances, we are ultimately guided by clinical judgment noting both a phenotype strongly in keeping with a desminopathy and an identified variant in the *DES* gene. Furthermore, extensive investigation has revealed no other likely explanation for her presentation.

Desmin myopathy is a very rare cause of cardiac arrhythmias. Close to 40 *DES* variants causing cardiac arrhythmias were identified up to 2022, of which 13 were associated with atrioventricular block, as in our case.^8^ Other arrhythmic manifestations include atrial fibrillation, bundle branch block, and arrhythmogenic right ventricular cardiomyopathy.^2^^,^^8^ Brodehl et al^9^ (2019) documented a similar case, with a 21-year-old man developing complete atrioventricular block requiring a permanent pacemaker. In that case, the variant was p.Y122H, and the investigators' functional analysis also showed protein aggregation. It remains uncertain if aggregation of desmin or the loss of its function is more relevant to progression of disease.^4^

Desmin myopathies unfortunately have no specific, definitive cure.^1^^,^^2^ Treatment is focussed on minimizing the impact of the various manifestations. Cardiomyopathy is treated in line with goal-directed medical therapy of heart failure, and atrioventricular block and arrhythmias with permanent pacemakers or implantable cardiac-defibrillators. However, research into macroautophagy stimulation, antioxidant treatment, and inhibition of certain cellular pathways may provide new avenues for therapeutic targets.^10^

## Conclusions

In summary, we present a case of a suspected novel desminopathy presenting initially with ventricular fibrillation and heart block and subsequently developing a cardiomyopathy and skeletal myopathy. Although the genetic report in isolation was classified as a VUS, we believe the variant was causative. Our case highlights the importance of determining the cause for unusual arrhythmic presentations, especially in young, previously well patients, and the increasing awareness of the wide spectrum of rare genetic conditions causing cardiac arrhythmias.

Our case also illustrates the challenges in diagnosing a desmin myopathy, given the wide range of potential clinical manifestations. Electromyography is used to exclude neurogenic causes but is not specific. Investigations such as electrocardiogram, echocardiography, and cardiac magnetic resonance will help identify conduction abnormalities and cardiomyopathy but will not definitively diagnose or exclude.^4^ Even muscle histology may not be sufficient in isolation to diagnose, given the presence of various other proteins within aggregates.^5^ This patient highlights the importance of careful follow-up and having a low threshold for multidisciplinary approach including neurology and genetic testing in assessing these patients, as extracardiac disease identification, which may occur decades after the initial cardiac event, is helpful in making the diagnosis.

## Funding Support and Author Disclosures

The authors have reported that they have no relationships relevant to the contents of this paper to disclose.

**Take-Home Messages**

• When assessing patients with unusual arrhythmic presentations, it is important to determine the cause, and we hope to increase awareness of genetic causes of cardiac arrythmias.
• Patients with desminopathy primarily involving the heart may develop extracardiac features decades later, and early multidisciplinary involvement is essential in optimizing management and improving patient outcomes.
