# Supplementary material for: Desminopathy: A Rare Cause of Cardiac Arrhythmia
Source: JACC Case Rep. 2026 Aug 10;31(41):109779. doi: 10.1016/j.jaccas.2026.109779 (PMC13689856; doi:10.1016/j.jaccas.2026.109779)
Supplement: Supplementary file 1 [file mmc1.docx]

Appendix A: List of genes included in genetic analysis

ACAD9, ACADM, ACADVL, ACTA1,ACTG2, ACTN2, ADCK3, AGL, ANO5, ATP2A1, B3GALNT2, B4GAT1, BAG3, BICD2, BIN1, BVES, C1QBP, CACNA1S, CAPN3, CASQ1, CAV3, CFL2, CHKB, CHST14, CLCN1, CNTN1, COL12A1, COL4A1, COL4A2, COL6A1, COL6A2, COL6A3, COQ2, CPT2, CRYAB, DAG1, DES, DGUOK, DMD, DNA2, DNAJB6, DNM2, DOK7, DPM3, DYSF, EMD, ENO3, ETFDH, FDX1L, FHL1, FKRP, FKTN, FLAD1, FLNC, GAA, GBE1, GFPT1, GMPPB, GOLGA2, GYG1, HNRNPA1, HNRNPDL, HSPB1, HSPB8, INPP5K, ISCU, ISPD, ITGA7, KBTBD13, KCNJ2, KLHL40, KLHL41, LAMA2, LAMP2, LARGE, LDB3, LDHA, LIMS2, LMNA, LMOD3, LPIN1, MAP3K20, MATR3, MEGF10, MICU1, MME, MPV17, MSTO1, MT-ATP6, MT-ATP8, MT-CO1, MT-CO2, MT-CO3, MT-CYB, MT-ND1, MT-ND2, MT-ND3, MT-ND4, MT-ND4L, MT-ND5, MT-ND6, MT-RNR1, MT-RNR2, MT-TA, MT-TC, MT-TD, MT-TE, MT-TF, MT-TG, MT-TH, MT-TI, MT-TK, MT-TL1, MT-TL2, MT-TM, MT-TN, MT-TP, MT-TQ, MT-TR, MT-TS1, MT-TS2, MT-TT, MT-TV, MT-TW, MT-TY, MTM1, MYH7, MYO18B, MYOT, MYPN, NEB, OPA1, ORAI1, PABPN1, PFKM, PGAM2, PGK1, PGM1, PHKA1, PHKB, PLEC, PNPLA2, POGLUT1, POLG, POLG2, POMGNT1, POMGNT2, POMK, POMT1, POMT2, PRKAG2, PUS1, PYGM, PYROXD1, RBCK1, RRM2B, RYR1, SCN4A, SELENON, SEPT9, SGCA, SGCB, SGCD, SGCG, SIL1, SLC22A5, SLC25A20, SLC25A4, SMCHD1, SMN1, SMN2, SMPX, SPEG, SPTBN4, STAC3, STIM1, SUCLA2, SUCLG1, SYNE1, SYNE2, TANGO2, TCAP, TIA1, TK2, TMEM126B, TMEM43, TNNT1, TNPO3, TOR1AIP1, TPM2, TPM3, TRAPPC11, TRIM32, TSFM, TTN, TTR, TYMP, VCP, VMA21, VPS13A YARS2. ACADM, ACTG2, ACTN2, AGL, BVES, C1QBP, CACNA1S, CHST14, CLCN1, CPT2, DNA2, DNM2, DOK7, ENO3, ETFDH, GFPT1, HNRNPA1, HSPB1, HSPB8, KCNJ2, LDHA, LPIN1, MATR3, MPV17, MYPN, OPA1, ORAI1, PFKM, PGAM2, PGM1, PRKAG2, PUS1, SLC25A4, SMPX, STIM1, SYNE2, TK2, TTR, VCP and YARS2

Appendix B: Initial DES variant interpretation report

| Gene | DES |
| --- | --- |
| Transcript | NM_001927.4 |
| Nomenclature | c.640-1G>C |
| Genotype | HET |
| Consequence | Splice acceptor variant |
| Inheritance | AD, AR |
| Classification | Likely pathogenic |
| Assembly | GRCh37/hg19 |
| POS | 2:220284972 |
| REF/ALT | G/C |
| gnomAD AC/AN | 0/0 |
| Polyphen | N/A |
| SIFT | N/A |
| MUTTASTER | Disease causing |
| Phenotype | Dilated cardiomyopathy (DCM),  Myopathy myofibrillar,  Scapuloperoneal syndrome neurogenic Kaeser type |
